# Real-time control of radiofrequency ablation using three-dimensional ultrasound echo decorrelation imaging in normal and diseased *ex vivo* human liver

**DOI:** 10.1088/1361-6560/adaacb

**Published:** 2025-02-05

**Authors:** Elmira Ghahramani, Peter D Grimm, Benjamin E Weiss, Nicholas S Schoenleb, Alexander J Knapp, Jiang Wang, Syed A Ahmad, Shimul A Shah, Ralph C Quillin, Sameer H Patel, T Douglas Mast

**Affiliations:** 1 Department of Biomedical Engineering, University of Cincinnati, Cincinnati, OH, United States of America; 2 Department of Pathology, University of Cincinnati, Cincinnati, OH, United States of America; 3 Department of Surgery, University of Cincinnati, Cincinnati, OH, United States of America

**Keywords:** Three-dimensional echo decorrelation imaging, real-time radiofrequency ablation control, human liver tissue, cirrhosis, steatotis, thermal tumor ablation

## Abstract

**Objective.:**

Ultrasound echo decorrelation imaging can successfully monitor and control thermal ablation of animal liver and tumor tissue *ex vivo* and *in vivo*. However, normal and diseased human liver has substantially different physical properties that affect echo decorrelation. Here, effects of human liver tissue condition on ablation guidance by three-dimensional echo decorrelation imaging are elucidated in experiments testing closed-loop control of radiofrequency ablation (RFA) in normal and diseased human liver tissue *ex vivo*.

**Approach.:**

Samples of normal, steatotic, and cirrhotic human liver tissue underwent RFA, targeting a 20 mm-diameter spherical ablation zone. For each tissue condition, RFA was controlled by echo decorrelation in N>14 trials, automatically ceasing if average cumulative decorrelation within the targeted ablation zone surpassed a predetermined threshold (successfully controlled trials), or otherwise completing a standard ablation cycle of the RFA generator (unsuccessfully controlled). For comparison, N=14 RFA trials for each tissue condition followed the RFA generator’s standard algorithm without echo decorrelation feedback (uncontrolled). Receiver operating characteristic (ROC) and precision-recall curve analyses compared 3D echo decorrelation maps to segmented ablation zones. To assess effects of closed-loop control and liver condition on treatment reliability, ablation volumes, rates, and Dice coefficients for measured vs. targeted ablation zones were statistically compared among control conditions and liver types.

**Results.:**

ROC curves showed effective prediction of local ablation by echo decorrelation across all liver types and control conditions (0.876 ⩽ AUROC ⩽ 0.953). Successful control was significantly more frequent, ablated volumes were generally larger, and optimal echo decorrelation thresholds were smaller for normal compared to diseased liver.

**Significance.:**

This study validates three-dimensional echo decorrelation imaging for monitoring and control of RFA in healthy and diseased human liver while elucidating the dependence of RFA and echo decorrelation outcomes on liver condition and resulting implications for clinical applications.

## 1. Introduction

Hepatocellular carcinoma (HCC) is the most common primary malignant tumor in the liver (Llovet *et al* 2021). Moreover, 25%–30% of patients diagnosed with colorectal cancer, the third leading cause of cancer death worldwide, develop liver metastases (Engstrand *et al* 2018). For many patients with malignant liver tumors, particularly small HCC or metastases from colorectal liver cancer, radiofrequency ablation (RFA) is an effective regional treatment (Lee and Lee 2018).

Because oncologic control in thermal ablation procedures depends on achieving a predefined coagulation zone, monitoring and control of the treatment progress in three dimensions (3D) is important to improve reproducibility and reliability of ablation treatments. Improved reproducibility of thermal ablation would improve outcomes by avoiding undertreatment, which can cause tumor recurrence, or overtreatment, which can result in injuries to critical structures such as biliary tract damage and perforation of the gastrointestinal tract (Howenstein and Sato 2010, Fonseca *et al* 2014, Geoghegan *et al* 2022).

Current methods for automatic control of RFA have limited capability. Some RFA probes incorporate integrated thermocouples or electrical impedance sensing, so that the RFA generator can use tissue temperatures or impedance at multiple locations as feedback to guide and control ablation (Zaltieri *et al* 2021). However, these single-point measurements do not provide information about the entire ablation zone and its surroundings. Magnetic resonance thermometry is capable of thermal ablation monitoring and control with update intervals on the order of a few seconds, but is expensive and vulnerable to motion artifacts, while also requiring specialized non-ferromagnetic ablation devices (Morikawa *et al* 2002, Napoli *et al* 2013, Ertürk *et al* 2016, Dong *et al* 2018, Hue *et al* 2018, Li *et al* 2021). Computed tomography (CT) is used primarily for treatment targeting and end-of-treatment evaluation and follow-up imaging, due to its use of ionizing radiation exposure and toxic contrast agents (Bruenn *et al* 2017, Inzerillo *et al* 2022). B-mode ultrasound imaging, a portable and inexpensive imaging modality with real-time capability (e.g. tens to hundreds of frames per second), has been utilized to monitor thermal ablation. B-mode imaging has also been employed to control RFA through its fusion with manually marked tumor zones on CT, MR, or contrast enhanced ultrasound images, using a stopping criterion of complete coverage of the targeted zone by a highly echoic area (Toshikuni *et al* 2017). However, B-mode imaging falls short in providing precise delineation of the ablation zone; for example, in many cases the ablated region can be isoechoic in comparison to the surrounding unablated tissue (Chiou *et al* 2007, Lee *et al* 2017, 2024, Zhi-yu *et al* 2017).

To overcome limitations of conventional B-mode imaging, pulse-echo ultrasound imaging methods have been developed for real-time feedback control in thermal ablation. One previously studied method is high-intensity focused ultrasound (HIFU) control using harmonic motion imaging (Curiel *et al* 2009) which can monitor heat-induced changes of tissue mechanical properties measured by acoustic radiation force-based elastography. Another method is temperature control with echo strain imaging, which aims to track position-dependent changes in echo arrival time caused by heat-induced temperature or elasticity changes (Casper *et al* 2012, 2013, Ebbini *et al* 2023). During thermal ablation, accuracy of these methods, which rely on cross-correlation of ultrasound echo signals to compute time delay, displacement, or strain, is adversely affected by decorrelation of the backscattered signals due to tissue motion, bubble activity, and heat-induced alterations in tissue structure (Curiel *et al* 2005, 2009, Kumon *et al* 2012, Casper *et al* 2013, Hooi *et al* 2015, Suomi *et al* 2016, Shi *et al* 2023)

Another approach to thermal ablation monitoring and control, ultrasound echo decorrelation imaging (Mast *et al* 2008, Kumon *et al* 2012, Subramanian *et al* 2014, Hooi *et al* 2015, Takagi *et al* 2016, Fosnight *et al* 2017, Abbass *et al* 2018a, 2018b, 2019, Cox *et al* 2020, Ghahramani *et al* 2022, Grimm 2022, Ghahramani *et al* 2024a), directly exploits the same heat-induced signal decorrelation that limits most correlation-based ultrasound monitoring approaches. In most implementations of this approach, echo signal decorrelation is computed between pairs of echo signals separated by milliseconds, then mapped to create quantitative 2D (Mast *et al* 2008, Subramanian *et al* 2014, Hooi *et al* 2015, Fosnight *et al* 2017, Abbass *et al* 2018a) or 3D (Ghahramani *et al* 2022, Grimm 2022) echo decorrelation images. These images directly quantify transient, heat-induced tissue structural changes such as protein denaturation, cell rupture, and tissue cracking, as well as heat-induced vapor and gas activity. Because echo decorrelation is proportional to the decoherence spectrum of local tissue reflectivity (Hooi *et al* 2015), echo decorrelation images provide a quantitative spatial map of these transient tissue structure changes that occur within millisecond time scales.

Ultrasound echo decorrelation imaging has shown promise for real-time control of thermal ablation (Takagi *et al* 2016, Abbass *et al* 2018a). HIFU and bulk ultrasound ablation in *ex vivo* bovine liver and *in vivo* rabbit liver and VX2 tumor have been successfully controlled (SC) using real-time, closed-loop 2D echo decorrelation feedback (Abbass *et al* 2018b, 2019). In bulk ultrasound ablation, unfocused ultrasound radiation is employed to heat a larger region compared to HIFU; resulting ablation rates are comparable to RFA and MWA (Subramanian *et al* 2014, Fosnight *et al* 2017, Abbass *et al* 2018b, 2019). In studies of thermal ablation control using echo decorrelation imaging, average or minimum cumulative echo decorrelation was computed within a target region of interest (ROI) and ablation was ceased after that value surpassed a predefined threshold ablation (Abbass *et al* 2018b, 2019, Ghahramani 2024c). Another recent study has validated control of RFA in *ex vivo* bovine liver using 3D echo decorrelation imaging (Grimm 2022).

However, although recent clinical studies have confirmed the promise of echo decorrelation for clinical thermal ablation monitoring (Ghahramani 2024c, Abbass *et al* 2023), echo decorrelation imaging of thermal ablation in human liver tissue has not previously been tested under controlled *ex vivo* conditions, and closed-loop control of thermal ablation using echo decorrelation imaging has not been studied in human tissue. These gaps in knowledge are important because heat-induced echo decorrelation may be expected to depend on the condition of human liver tissue, including both normal and diseased liver parenchyma which have significantly different acoustic, thermal, and physicochemical characteristics (Ghahramani 2024c, Ghahramani 2022). HCC usually occurs in cirrhotic liver, where tumor resection is usually contraindicated due to limited hepatic reserve and thermal ablation is considered the best available treatment (Allaire *et al* 2020, Bengtsson *et al* 2022). HCC can also develop *de novo* in patients with non-alcoholic fatty liver disease and non-alcoholic steatohepatitis (Dhamija *et al* 2019). In contrast, metastatic liver tumors can occur within a background of normal liver parenchyma. These different liver tissue conditions are likely to affect performance of thermal ablation and its guidance by ultrasound imaging; for example, cirrhosis has been shown to significantly affect ablation zone size, so that thermal ablation parameters may need to be adjusted accordingly (Ding *et al* 2023). For effective use of this promising ablation monitoring and control approach, including translation to clinical applications, better understanding is needed regarding the dependence of these ablation and imaging effects on the physical properties of liver tissue, which depend directly on its disease conditions.

In this study, we evaluate monitoring and control of RFA using 3D echo decorrelation imaging in normal and diseased human liver *ex vivo*. A secondary goal was to compare ablation outcomes for *ex vivo* RFA of normal and diseased human liver tissue. The performance of 3D echo decorrelation monitoring and control for RFA is analyzed in normal, steatotic, and cirrhotic liver parenchyma *ex vivo*. Through statistical comparison of control success, ablation outcomes, and ablation zone prediction among these tissue conditions, including statistical modeling of ablation outcomes using liver tissue condition and echo decorrelation as independent variables, this work provides new insight into the dependence of echo decorrelation, RFA outcomes, and the potential for closed-loop control on the physical condition of human liver tissue.

## 2. Methods

### 2.1. Tissue collection

Specimens of human liver tissue were obtained from organs explanted before liver transplant, from autopsy specimens, and from organs declined for transplant. The process for tissue acquisition, including consent from all living tissue donors, was performed under protocols approved by the University of Cincinnati Institutional Review Board (UC IRB protocols 2014–4755 and 2020–0883).

The study included 54 subjects, from which 8 subjects provided the samples used in preliminary trials (N=12) and 46 subjects provided the samples used in primary trials (N=97). Information on the samples used in these trials is summarized in table 1. The medical team involved with liver transplant, including a pathologist co-author, diagnosed tissue conditions of the explanted liver tissue. For autopsy and declined specimens, diagnoses were determined through pathology or CT imaging conducted and reported by an involved physician. Liver conditions spanned from normal, characterized by less than 10% fat infiltration, to steatotic, exhibiting more than 10% fat infiltration (Salt 2004, de Lédinghen *et al* 2014, Son *et al* 2016), to cirrhotic with extensive, severe scar tissue. Before ablation experiments, each sample was visually assessed for its degree of stiffness, yellowing, and scar tissue appearance; observations were found to be consistent with diagnosis for each specimen.

Liver specimens were cut into approximately 60 × 60 × 60 mm^3^ cubes and stored in a −80 °C freezer. From each liver specimen, 1–6 cubic samples were used, distributed into blocks of N=14 uncontrolled (UC) trials and N≥14 control attempts for each of the three liver tissue conditions.

### 2.2. RFA experiments

**Figure 1.**
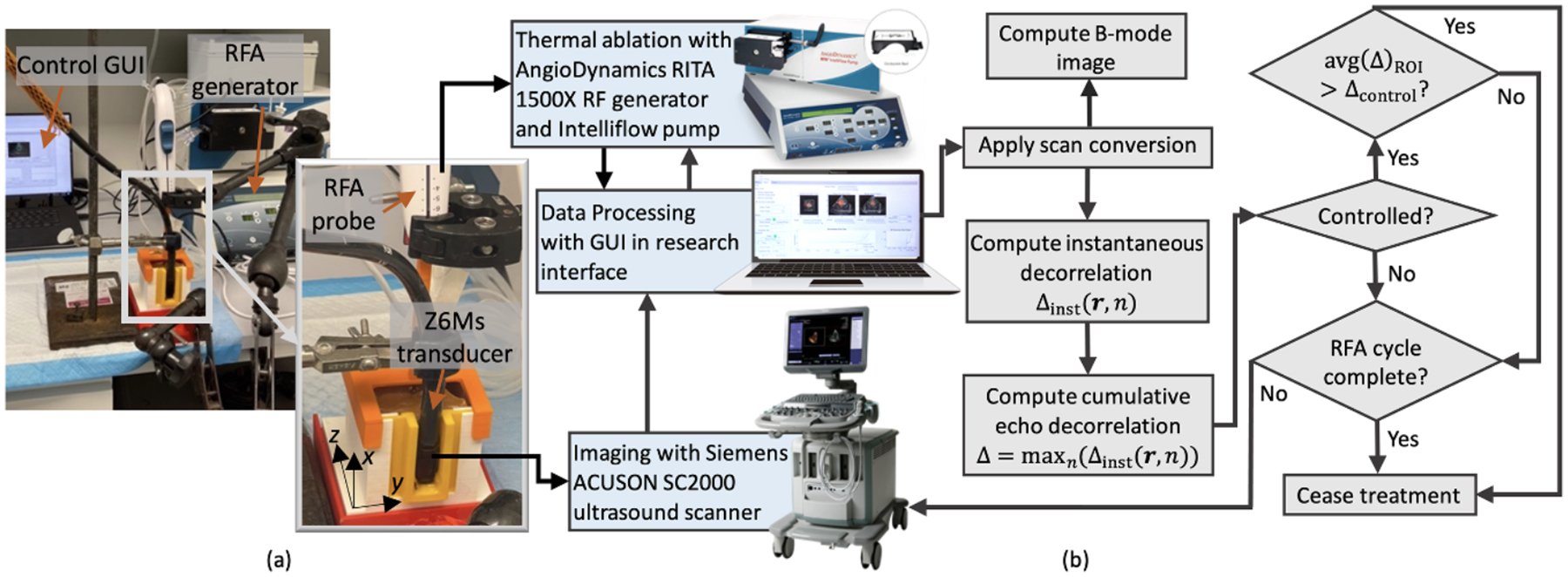
Experimental setup and echo decorrelation (Δ) feedback control algorithm for *ex vivo* human liver RFA control. (a) Experimental setup: liver tissue placed inside the white cuvette, RFA needle inserted through a guide built into the orange lid, and Z6Ms transesophageal ultrasound array fixed in a Tegaderm-covered window using the yellow 3D-printed window cover. Azimuth (y), range (z), and elevation (x) directions are shown in yellow Cartesian coordinates. (b) Control algorithm flow chart.

Before *ex vivo* ablation experiments, specimens were retrieved from the freezer, placed in phosphate-buffered saline (PBS 0.01 M, pH 7.4 at 25 °C, Sigma Life Science P3813, St. Louis, MO, USA), and thawed for 1 d in a laboratory refrigerator. The experimental setup was the same as a previous preliminary study (Ghahramani *et al* 2021, Ghahramani 2024c) and is shown in figure 1. Each specimen was placed in a 3D-printed 60 × 60 × 60 mm^3^ cuvette (printed using polylactic acid filament), with an acoustic window covered by a Tegaderm^™^ film adhesive membrane (3 M Health Care, St. Paul, MN, USA). The cuvette was then filled with room-temperature PBS to fill any air gaps within the tissue, and the tissue was allowed to acclimate to room temperature. The cuvette containing liver tissue was placed on a 3D-printed stand, and a transesophageal phased array transducer (Z6Ms, Siemens Medical Systems, Erlangen, Germany) was fixed within a fitted holder to face the liver tissue through the acoustic window (figure 1). An RFA needle (RITA StarBurst XLi-enhanced device with micro-infusion, AngioDynamics, Manchester, GA, USA) was then inserted into the tissue through a needle guide integrated into a 3D-printed cuvette lid. This design reproducibly placed the RFA tip 30 mm from the cuvette bottom and side walls, centered in the azimuth and elevation directions of the acquired ultrasound image at about 30 mm range, with an estimated placement uncertainty of 1–2 mm.

RFA exposure parameters were adapted from the manufacturer’s Instructions for Use document (AngioDynamics^®^ 2010), which specifies standard clinical protocols for tissue ablation using this RFA generator and probe. Matching standard clinical protocols for ablation of a 20 mm diameter volume of liver or soft tissue, the RFA needle was deployed to a tine diameter of 20 mm and the RFA generator (RITA 1500X RF Generator, AngioDynamics, Manchester, GA, USA) was programmed with a target temperature of 105 °C. Based on previous *ex vivo* experimental results and to account for the lack of tissue perfusion in *ex vivo* ablation, RFA power was set to 50 W, while the duration for tissue to be held at target temperature was programmed as 3 min. Preliminary testing confirmed that these exposure conditions resulted in ablation of an approximately 20 mm diameter spherical ablation zone, centered at the needle tip and contained completely within the ultrasound image volume. During ablation, saline infusion was applied using the RFA generator’s matching pump device (Intelliflow pump, AngioDynamics, Manchester, GA, USA), to maintain tissue conductivity and reduce tissue charring by cooling the vicinity of the needle’s active tines, as specified in the clinical protocol for liver or soft tissue ablation using this system (AngioDynamics^®^ 2010).

After each ablation procedure, tissue was frozen inside the same cuvette at −80 °C for at least 12 h. Tissue was then retrieved, partially defrosted for removal from the cuvette, and sectioned using a commercial meat slicer at a uniform thickness of 3–4 mm. In cases where ablation zone boundaries were not visually clear in diseased liver tissue, tissue sections were vitally stained with a 2% (w/v) solution of triphenyl tetrazolium chloride (TTC, Sigma-Aldrich, St. Louis, MO, USA) in 0.01 M PBS for 20–30 min. Tissue sections were then optically scanned at 600 dpi (V550, Epson America, Inc. Long Beach, CA, USA). Using MATLAB (The MathWorks, Natick, MA, USA), each tissue section was cropped to a square matching the cuvette’s cross section, accounting for the boundaries where tissue contacted the cuvette’s walls, and was then manually segmented based on discoloration of ablated vs. unablated regions. The 3D ablation zone was then reconstructed by registering each segmented 2D section to its correct elevational position in the cuvette, similar to previous studies (Ghahramani *et al* 2021, 2022).

### 2.3. Ultrasound echo decorrelation imaging

Throughout each ablation procedure, pulse-echo image volumes were acquired at a center frequency of 4.5 MHz and an imaging depth of 70 mm using an Acuson SC2000 scanner and Z6Ms matrix array (Siemens Medical Systems, Erlangen, Germany). Each echo volume comprised 85 × 67 complex, demodulated in-phase/quadrature (IQ) scan lines, spanning 90° × 90° in the azimuth and elevation directions, respectively. Each scan line comprised 485 samples of IQ echo data recorded at a sampling rate of 5.33 MHz, sufficient to fully capture the demodulated signal bandwidth of about 5 MHz. In this configuration, the system’s volumetric frame rate was 20 volumes per second.

Echo decorrelation imaging tracks millisecond-scale, heat-induced transient changes between sequential complex echo volumes (Mast *et al* 2008, Gudur *et al* 2012, Kumon *et al* 2012, Subramanian *et al* 2014, Abbass *et al* 2018a, 2018b, Zhou *et al* 2019, Ghahramani *et al* 2022). To compute a 3D echo decorrelation image between two sequential image volumes, echo volumes were acquired in pairs, with each pair comprising two echo volumes separated by the inter-frame time of 50 ms. Pairs of image volumes were then transferred by a gigabit local Ethernet connection to a laptop computer (Dell Precision 5510 with an Intel Core-i7 6820HQ processor) running a custom graphical user interface (GUI) that computed and displayed echo decorrelation images, while also interfacing with the Angiodynamics RFA generator for controlled ablation trials. Due to time requirements for data storage, transfer, and decorrelation computation, echo volume pairs were captured once every 22 s throughout each RFA procedure.

All processing of image data was performed in MATLAB (The MathWorks, Natick, MA). For computation of echo decorrelation images, IQ image volumes were scan-converted to a Cartesian grid spanning 60 × 60 × 60 mm^3^ with an isotropic step size of 0.5 mm in each direction, similarly to a previous study (Ghahramani *et al* 2022). Combined-normalized 3D echo decorrelation per millisecond *et al* was computed as described previously (Ghahramani *et al* 2022),

$$\Delta_{\text{inst}}(r,n)=2\left[\frac{\beta^{2}(r,n)-{\left|R_{01}(r,n)\right|}^{2}}{\tau \left[\beta^{2}(r,n)+\bar{\beta^{2}}(n)\right]}\right]$$

where temporal indices n sequentially number each acquired echo volume pair, r is a position vector within an echo volume, $R_{01}(r,n)$ is the zero-lag, spatially windowed cross-correlation between the two sequential complex echo volumes comprising each pair, $\beta^{2}(r,n)$ is the product of zero-lag, spatially windowed autocorrelations for sequential echo volumes, and $\bar{\beta^{2}}(n)$ is the spatial average of $\beta^{2}(r,n)$ within the cubic volume. Spatial windows employed for cross-correlation and autocorrelation were isotropic, 3D Gaussian functions with width parameter σ=3mm. The resulting decorrelation maps are near zero at locations where consecutive images do not substantially differ, and largest where they differ the most. One cumulative decorrelation (Δ) map per ablation trial was generated by taking the temporal maximum decorrelation at each position r.

For displaying decorrelation maps, duplex B-mode/Δ images were constructed by overlaying the grayscale B-mode image with a parametric image of the log_10_-scaled Δ with transparency proportional to the logarithmically scaled Δ, mapped over a defined dynamic range. B-mode images were constructed from logarithmically scaled envelopes $20\cdot {\text{log}}_{10}\left(\left|I_{0}(r)\right|\right)$ of IQ scan lines from the first imaged volume of each acquired pair and displayed using a 65 dB dynamic range.

### 2.4. Feedback control algorithm

A real-time echo decorrelation imaging feedback algorithm (figure 1(b)) was used for controlling RFA in *ex vivo* human liver. This algorithm was implemented in a custom GUI designed in MATLAB, similar to a previous study in RFA control of the *ex vivo* bovine liver (Grimm 2022). Compared to the previous study (Grimm 2022), here a matrix array operating at a higher frequency (4.5 MHz), similar to ultrasound arrays used for intraoperative imaging, was employed. By use of this higher-frequency transducer, the manufacturer-estimated axial and lateral resolution of B-mode images were improved from 0.7 and 2.2 mm, respectively, in the previous control study (Grimm 2022) to 0.3 and 1.7 mm in the present study. During ablation, a 3D cumulative echo decorrelation map was computed as the temporal maximum of the instantaneous echo decorrelation images $\left(\Delta_{\text{inst}}\right)$ up to the current time point and displayed within the GUI as three perpendicular cross-sections of echo decorrelation superimposed on current B-mode images.

For trials controlled by the generator’s default algorithm but not controlled by echo decorrelation feedback, called uncontrolled (UC) trials here (N=14 normal liver, N=14 steatotic liver, N=14 cirrhotic liver), ablation proceeded until the average temperature of three integrated thermocouples reached the target temperature of 105 °C and remained there for 3 min. For trials controlled by echo decorrelation imaging, called ‘controlled trials’ here (N=16 normal liver, N=24 steatotic liver, N=15 cirrhotic liver), samples were ablated using the same RFA parameters and Δ was computed in real time within a spherical region of interest (control ROI) with diameter 20 mm centered at the tip of RFA needle, corresponding to the targeted ablation zone. If the spatial average of within the control ROI surpassed a prespecified threshold $\Delta_{\text{control}}$, treatment was automatically ceased through a custom-designed microcontroller circuit between the laptop and the RFA generator and the trial was considered successfully controlled (SC). If this threshold was not reached, ablation continued until completion of the RFA generator’s cycle and the trial was considered ‘unsuccessfully controlled’ (USC). There were thus three control conditions considered: UC, SC, and USC.

The control threshold was found through a set of preliminary RFA trials (Ghahramani *et al* 2021), including 6 UC trials (3 normal, 1 steatotic, and 2 cirrhotic liver parenchyma specimens) and 6 controlled ablation trials with varying thresholds (log_10_-scaled decorrelation per ms) between −2.8 and −2.3 (1 normal, 1 steatotic, and 4 cirrhotic liver parenchyma specimens). Final average cumulative decorrelation within the 20 mm diameter spherical control ROI was found to be −2.49 ± 0.38 (mean ± standard deviation log_10_-scaled decorrelation per ms) for these 12 preliminary trials and exceeded −2.8 in all preliminary UC trials and 4 of 6 preliminary controlled trials. The cutoff point −2.8 (log_10_-scaled decorrelation per ms) corresponded to 90% specificity for prediction of local ablation by echo decorrelation, computed as TN/(TN + FP), where TN and FP denote true negative and false positive predictions, respectively. Increased specificity results from a decrease in false-positive predictions, meaning fewer incorrect predictions of unablated locations as ablated. In clinical RFA, reduced FP predictions could enable a reduced rate of incomplete treatment, potentially reducing local tumor recurrence. Consistent with previous studies where the control threshold matched 90% specificity for local abation prediction (Fosnight *et al* 2017, Abbass *et al* 2018b), −2.8 (log_10_-scaled decorrelation per ms) was chosen as the control threshold for this study.

### 2.5. Data analysis

To evaluate dependence of ablation control success rates on liver tissue condition, proportions of SC trials versus total number of control attempts were determined separately for normal, steatotic, and cirrhotic liver tissue. Rates of control success were then compared between tissue conditions using a two-tailed Chi-squared test. The significance criterion employed for this and all other statistical tests was p<0.05.

To assess local prediction of thermal ablation, 3D echo decorrelation images were compared with corresponding tissue histology using receiver operating characteristic (ROC) (Mast *et al* 2008, Subramanian *et al* 2014, Grimm 2022) and precision-recall (PR) curve analysis (Saito and Rehmsmeier 2015, Jones *et al* 2020). An ROC curve plots sensitivity or true positive rate (TPR) versus 1 − specificity or false positive rate (FPR), while a PR curve plots the precision or positive predictive value (PPV) versus recall or sensitivity (TPR). 3D ablation volumes reconstructed from manually segmented tissue slices were compared voxel-by-voxel with 3D echo decorrelation maps for the three tissue conditions (normal, steatotic, and cirrhotic liver) and three control conditions (UC, SC, and USC), resulting in nine ROC and nine PR curves. All ROC and PR computations were performed within a spherical ROI of radius 25 mm, comprising the control ROI and a 15 mm surrounding margin, and completely encompassed by the echo volume.

Areas under ROC curves (AUROC) were compared statistically versus the null hypothesis (AUROC = 0.5, one tailed) and each other (two tailed, across control conditions for each liver type and across liver types for each control condition) using a Z test employing a general model for AUC standard error (Hanley and McNeil 1982, Krzanowski and Hand 2009), implemented using the pROC package in R (R Foundation for Statistical Computing, Vienna, Austria) (Robin *et al* 2011). To perform a similar Z test for differences in area under PR curves (AUPR), Z scores were computed in R using the bootstrap method with 5000 sample draws (Zobolas 2021). To ensure meaningful comparisons, the region under each PR curve corresponding to the minimum achievable AUPR was accounted for (Boyd *et al* 2012). The area of this region, computed based on the class skew of π=(ablatedvoxels)/(allvoxels), can be neglected if the skew is large (π⩽0.1) (Boyd *et al* 2012), which was the case for all nine groups in this study (π<0.07).

Z statistics for prediction assessments by both AUC and PR curves were scaled to account for the effective number of independent ablation predictions $N_{\text{eff}}$ vs. the total number of predicted voxels $N_{\text{tot}}$ using the factor $\sqrt{N_{\text{eff}}/N_{\text{tot}}}={\left(\sqrt{2}(l/d{)}^{3}\right)}^{1/2}$, as described previously (Subramanian *et al* 2014, Ghahramani *et al* 2022). In this equation, l=0.5mm is the isotropic step size of the Cartesian grid and d=2.355σ is the full width at half maximum of a Gaussian distribution with σ=3mm, representing the window width parameter used in the echo decorrelation computations (Ghahramani *et al* 2022). Effective p values for prediction assessments were determined from these scaled Z statistics using the cumulative distribution function of the standard normal distribution.

Optimal thresholds for ablation prediction based on ROC curves were chosen as the point maximizing the difference between TPR and FPR, corresponding to a maximum of Youden’s statistic J=TPR-FPR. For PR curves, optimal prediction thresholds were chosen as the point maximizing the $F_{1}$-score, equal to the harmonic mean of PPV and TPR, i.e. ${\text{F}}_{1}=2*\text{PPV}*\text{TPR}/(\text{PPV}+\text{TPR})$. Notably, the $F_{1}$ score is equivalent to a Dice coefficient characterizing agreement between predicted and measured ablation zones.

Echo decorrelation cutoffs corresponding to 90% specificity for local ablation prediction, which can be considered candidate thresholds for use in control of RFA by 3D echo decorrelation imaging, were computed separately for normal, steatotic, and cirrhotic liver tissue. For each tissue type, these cutoffs were computed across all primary trials (*N* = 30 normal liver, *N* = 38 steatotic liver, *N* = 29 cirrhotic liver).

The effect of liver tissue condition, control condition, and subject gender on ablation and imaging outcomes were statistically evaluated based on four outcome variables: the measured ablated volume, ablation rate, Dice coefficients for measured vs. targeted 3D ablation regions, and final average cumulative decorrelation within the control ROI. To ensure appropriate comparisons, normality of all variables was first confirmed using the Shapiro–Wilk test and homogeneity of variances was evaluated using the Bartlett test (Bartlett 2018b). For groups without significantly different variances, the four analyzed outcome variables were then compared by three-way ANOVA to evaluate the null hypothesis of equal means, followed by a *post hoc* Tukey’s honest significance difference test performed on the significant ANOVA results (groups without significantly different variances). For groups with significantly different variances, a three-way Kruskal–Wallis rank-sum test was employed on the same outcome variables to assess the hypothesis of no median difference (Kruskal and Wallis 1952), followed by Dunn’s test with Bonferroni adjustment performed on significant Kruskal–Wallis results (Dunn 1964). Statistical significance of differences in variance were assessed using a two-sample F-test.

Relations between echo decorrelation and ablation outcomes were assessed across all control conditions and tissue types using linear regression. In this modeling, lines of best fit were determined between the overall echo decorrelation (final log_10_-scaled average cumulative decorrelation in the spherical control ROI with 20 mm diameter) vs. the segmented ablation volume (ml), ablation rate (ml min^−1^), and Dice coefficient between targeted and measured ablation regions. Statistical significance and goodness of fit were assessed using the Pearson correlation coefficient and RMS error between modeled and measured outcomes.

To further elucidate the effect of tissue condition on echo decorrelation and on RFA outcomes, multiple linear regression models were constructed for the same three ablation outcomes (ablation volume, ablation rate, and Dice coefficient of measured vs. targeted ablation regions), using tissue condition and final log_10_-scaled, ROI-average cumulative echo decorrelation as independent (input) variables. Models were implemented using the MATLAB function *regress*. Tissue condition was represented using binary categorical variables for steatosis and cirrhosis. Goodness of fit was assessed using the Pearson correlation coefficient and RMS error between modeled and measured outcomes. Statistical significance was assessed based on an F test for a linear relationship between input and output variables.

## 3. Results

For controlled trials in normal liver, the average cumulative echo decorrelation within the control ROI surpassed the control threshold of −2.8 (log_10_-scaled decorrelation per ms) in 14 of 16 trials (87.5%), which were considered SC. In contrast, 11 of 24 controlled trials in steatotic liver (45.8%) and 6 of 15 trials in cirrhotic liver (40.0%) surpassed this echo decorrelation threshold and were considered SC. The control success rate for normal liver tissue was significantly higher than for steatotic and cirrhotic liver, based on the Chi-squared test (p=0.0085 and $p=6.6\times 10^{-3}$, respectively). There was no significant difference between control success rates for steatotic vs. cirrhotic liver (p>0.05).

**Figure 2.**
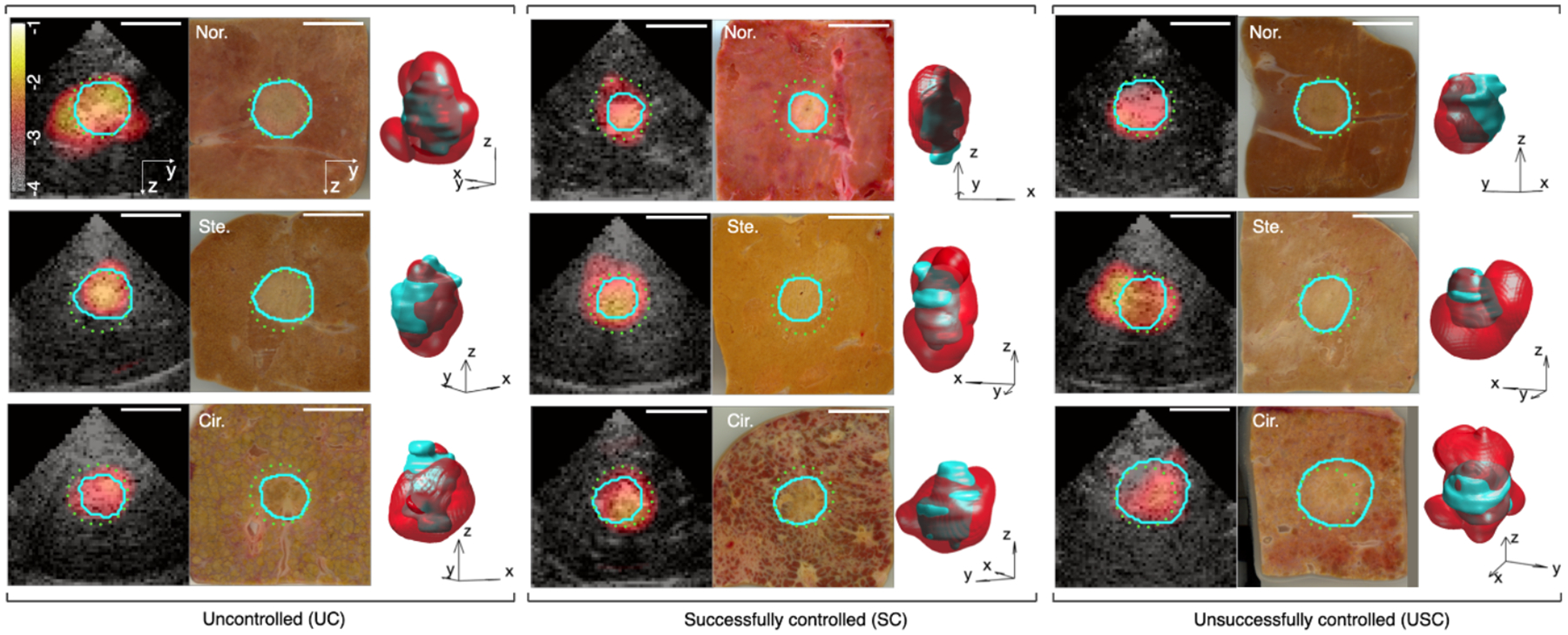
B-mode and echo decorrelation images and corresponding tissue histology for nine representative trials for the three control conditions and three liver types. Each row corresponds to a liver tissue condition, normal (Nor.), steatotic (Ste.), or cirrhotic (Cir.). Groups arranged from left to right represent uncontrolled (UC), successfully controlled (SC), and unsuccessfully controlled (USC) trials respectively. The color bar at the top left sub-figure ranges from −4 to −1 (log_10_-scaled decorrelation per ms). White scale bars indicate 20 mm. For each trial, a grayscale B-mode image cross-section is overlaid with an echo decorrelation (‘hot’ colormap) at elevation zero, the corresponding tissue section closest to this elevation is shown with its manually segmented ablation zone (cyan boundaries), and 3D echo decorrelation iso-surfaces are plotted at 10^−3.2^ (log_10_-scaled decorrelation per ms) in red with the 3D ablation zone in cyan, rotated for convenience of 3D visualization. The Cartesian coordinate axes denote elevation (x), azimuth (y), and range (z). The 20 mm-diameter control ROI boundary is plotted as a dotted green circle in the cross-sectional image panels.

Figure 2 illustrates the results of 3D echo decorrelation imaging for nine trials, representing typical outcomes of ablation zones and decorrelation maps. The three rows display RFA procedures in normal, steatotic, and cirrhotic liver, while the three columns present representative results for UC, SC, and USC trials, respectively. For each trial represented, three sub-panels show a grayscale B-mode image overlaid with a parametric echo decorrelation image at elevation zero, the corresponding segmented tissue section closest to elevation zero, and a 3D iso-surface of echo decorrelation at 10^−3.2^ ms^−1^ plotted together with the 3D reconstructed ablated zone. Generally good correspondence is seen between areas of elevated echo decorrelation and segmented ablation zones, with best correspondence in the displayed SC and USC trials for normal liver, UC and SC trials for steatotic liver, and UC trials for cirrhotic liver. Comparing between control conditions (figures 2(a)–(c)), best correspondence between the decorrelation map and the ablation zone is seen in steatotic and cirrhotic liver tissue for UC trials (panel (a)) and in normal liver for both SC and USC trials (panels (b) and (c)). These were typical observations across all trials in this study.

**Figure 3.**
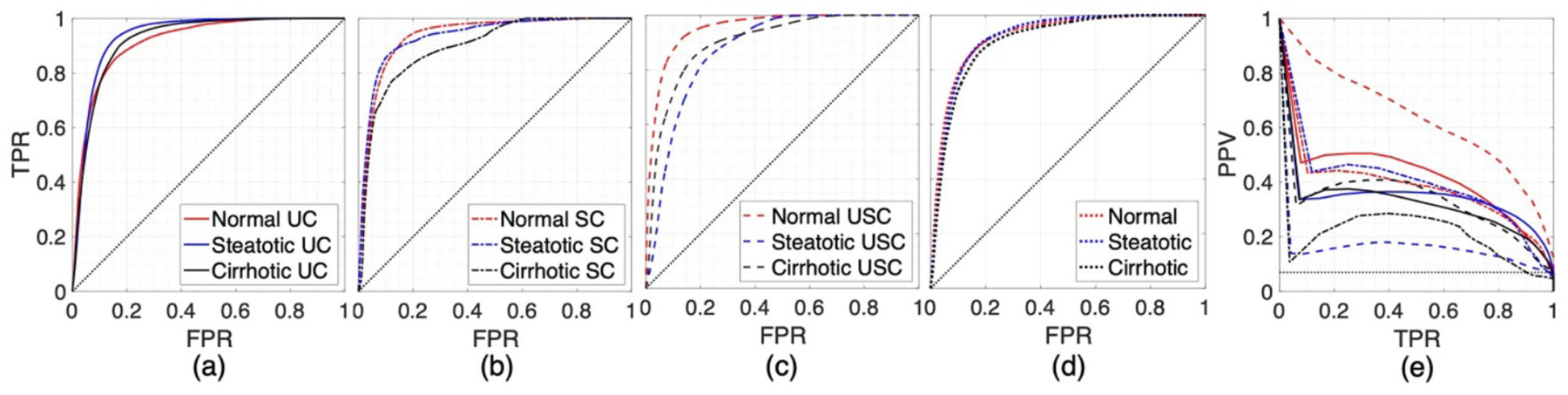
ROC and PR curves representing the predictive performance of 3D echo decorrelation for RFA in healthy and diseased *ex vivo* human liver. (a) ROC curves for uncontrolled (UC) trials. (b) ROC curves for successfully controlled (SC) trials. (c) ROC curves for unsuccessfully controlled (USC) trials. (d) ROC curves for all three liver tissue conditions across all three control conditions. (e) PR curves for the nine groups resulting from the combination of three control condition and three liver tissue conditions, with legends matching panels (a)–(c). * p<0.05, ** p<0.01, *** p<0.001.

Figure 3 presents ROC and PR curves constructed from direct comparison between the 3D echo decorrelation maps and corresponding 3D reconstructed ablation zones, while corresponding AUROC and AUPR are listed in table 2. ROC curves for UC, SC, and USC trials are shown in figures 3(a)–(c), respectively, with separate curves for normal, steatotic, and cirrhotic liver conditions. Figure 3(d) displays ROC curves computed across all three control conditions for each liver type; all nine ROC curves demonstrated high AUROC values (⩾ 0.876). Figure 3(e) shows the nine corresponding PR curves for the same combinations of three control conditions and three tissue conditions as shown in figures 3(a)–(c).

Cutoff points corresponding to $J_{\text{max}}$ and $F_{1\text{max}}$, from ROC and PR curves computed over all primary trials combined within each of the nine groups, are also reported in table 2. Optimal thresholds maximizing J were generally smaller than those maximizing $F_{1}$. The cutoff points corresponding to the point nearest the top left-hand corner of the ROC plot, which simultaneously maximizes sensitivity and specificity (Krzanowski and Hand 2009), were comparable to the corresponding cutoff point of −3.71 (log_10_-scaled decorrelation per ms) found in a previous study of 3D echo decorrelation imaging of *ex vivo* bovine liver RFA (Ghahramani *et al* 2022), and were within the range ±0.15 (log_10_-scaled decorrelation per ms) from optimal thresholds maximizing the J index.

In comparisons of echo decorrelation prediction performance between liver tissue conditions, performed separately for each control condition, AUROC values were not significantly different. In comparisons of PR curves for UC trials, prediction of local ablation for normal liver (AUPR = 0.423) was significantly better than for cirrhotic liver (AUPR = 0.328, p=0.0222), while no significant differences were seen for normal or cirrhotic liver vs. steatotic liver. For SC trials, echo decorrelation predicted ablation significantly better for normal liver (AUPR = 0.387) and steatotic liver (AUPR = 0.399) than for cirrhotic liver (AUPR = 0.214, $p=4.62\times 10^{-4}$ and $p=9.52\times 10^{-4}$ respectively). For USC trials, local ablation prediction by echo decorrelation was significantly better for cirrhotic liver (AUPR = 0.335) than for steatotic liver (AUPR = 0.164, $p=1.08\times 10^{-6}$).

Comparisons between ROC curves between control conditions, performed separately for each tissue type, showed no significant differences in prediction of local ablation by echo decorrelation imaging in most cases, except that steatotic UC trials and SC trials showed significantly better ablation prediction than steatotic USC trials for both ROC curve analysis (AUROC = 0.936 and 0.934 vs. 0.876, $p=3.21\times 10^{-6}$ and $p=2.99\times 10^{-3}$ respectively) and for PR curve analysis (AUPR = 0.350 and 0.399 vs. 0.164, $p=1.04\times 10^{-9}$ and $p=8.02\times 10^{-8}$ respectively). PR curves also indicated that for cirrhotic liver tissue, UC and USC trials showed better ablation prediction than SC trials (AUPR = 0.328 and 0.335 vs. 0.214, $p=7.91\times 10^{-3}$ and p=0.0121 respectively).

**Figure 4.**
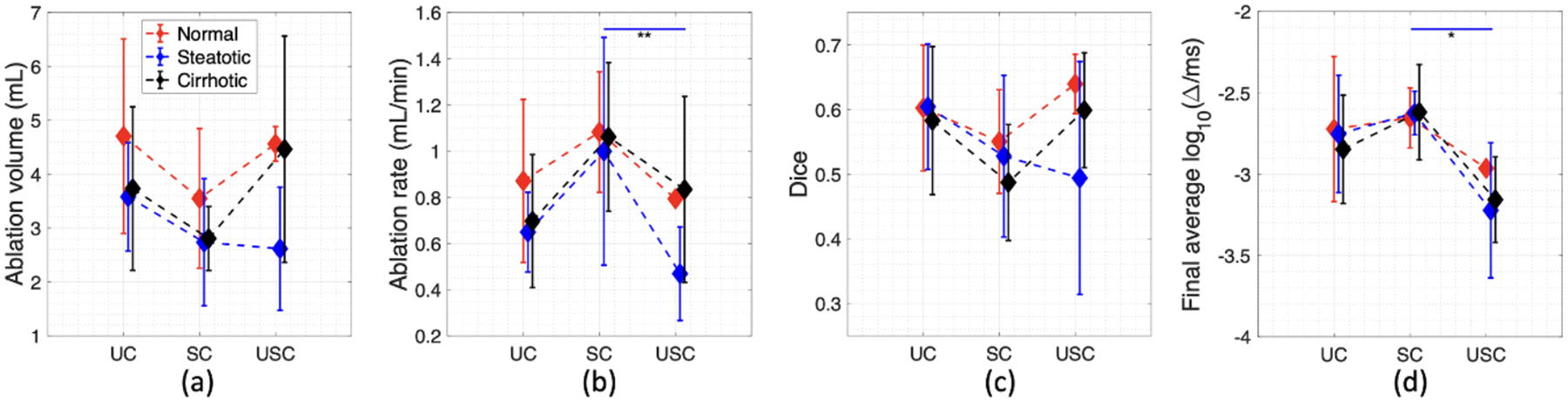
Ablation outcomes for uncontrolled (UC), successfully controlled (SC), and unsuccessfully controlled (USC) trials in normal (red), steatotic (blue) and cirrhotic (black) liver tissue. (a) Ablation volume (ml). (b) Ablation rate (ml min^−1^). (c) Dice coefficient comparing measured versus targeted ablation zones. (d) Final cumulative decorrelation averaged within the spherical control ROI with diameter 20 mm. *: p<0.05, **: p<0.01.

Figures 4(a)–(d) and table 3 show means and standard deviations of the measured ablated volume (ml), ablation rate (ml min^−1^), Dice coefficient comparing the ablation zone versus the control ROI (target ablation region), and final log_10_-scaled average decorrelation inside the control ROI for each group. In figure 4, these results are plotted grouped by control condition, with liver tissue condition indicated by red lines for normal, blue for steatotic, and black for cirrhotic liver tissue. All these parameters fulfilled normality criteria according to the Shapiro–Wilk test (p>0.05), while homogeneity of variance criteria, evaluated using the Bartlett test, were met for all parameters (p>0.05) except for ablation rate (p=0.0033). As shown in figure 4(a), average ablation volumes for SC trials with all three liver tissue conditions were generally smaller than for corresponding UC trials, while normal liver tissue incurred higher average ablated volume than cirrhotic or steatotic liver tissue for each control condition; however, these differences were not statistically significant (p>0.05). Ablation rates for all three liver tissue conditions, shown in figure 4(b), were largest for SC trials and were generally similar between UC and USC trials. For steatotic liver tissue, the average ablation rate was significantly higher for SC than for USC trials (Kruskal–Wallis rank-sum test, p=0.005), while ablation rate variance was significantly larger for SC trials compared to UC and USC trials (p<0.001).

Some significant differences were seen between ablation rate variance for different tissue conditions, including significantly higher variance for normal vs. steatotic liver tissue in UC trials (p=0.0148), for steatotic vs. normal liver tissue in SC trials (p=0.0353), and for cirrhotic vs. steatotic liver tissue in USC trials (p=0.0328). Figure 4(c) shows moderate agreement between measured and targeted ablation zones for all the control and tissue conditions (0.457 ⩽ Dice ⩽ 0.672), with differences that were not statistically significant (p>0.05). As seen in figure 4(d), the final log_10_-scaled, spatially averaged cumulative echo decorrelation within the control ROI was lowest for USC trials, a significant difference for trials with steatotic liver tissue compared to corresponding SC trials (p=0.0127). In three-way ANOVA tests, gender did not show a significant effect on any of the parameters summarized in figure 4 (p>0.05).

**Figure 5.**
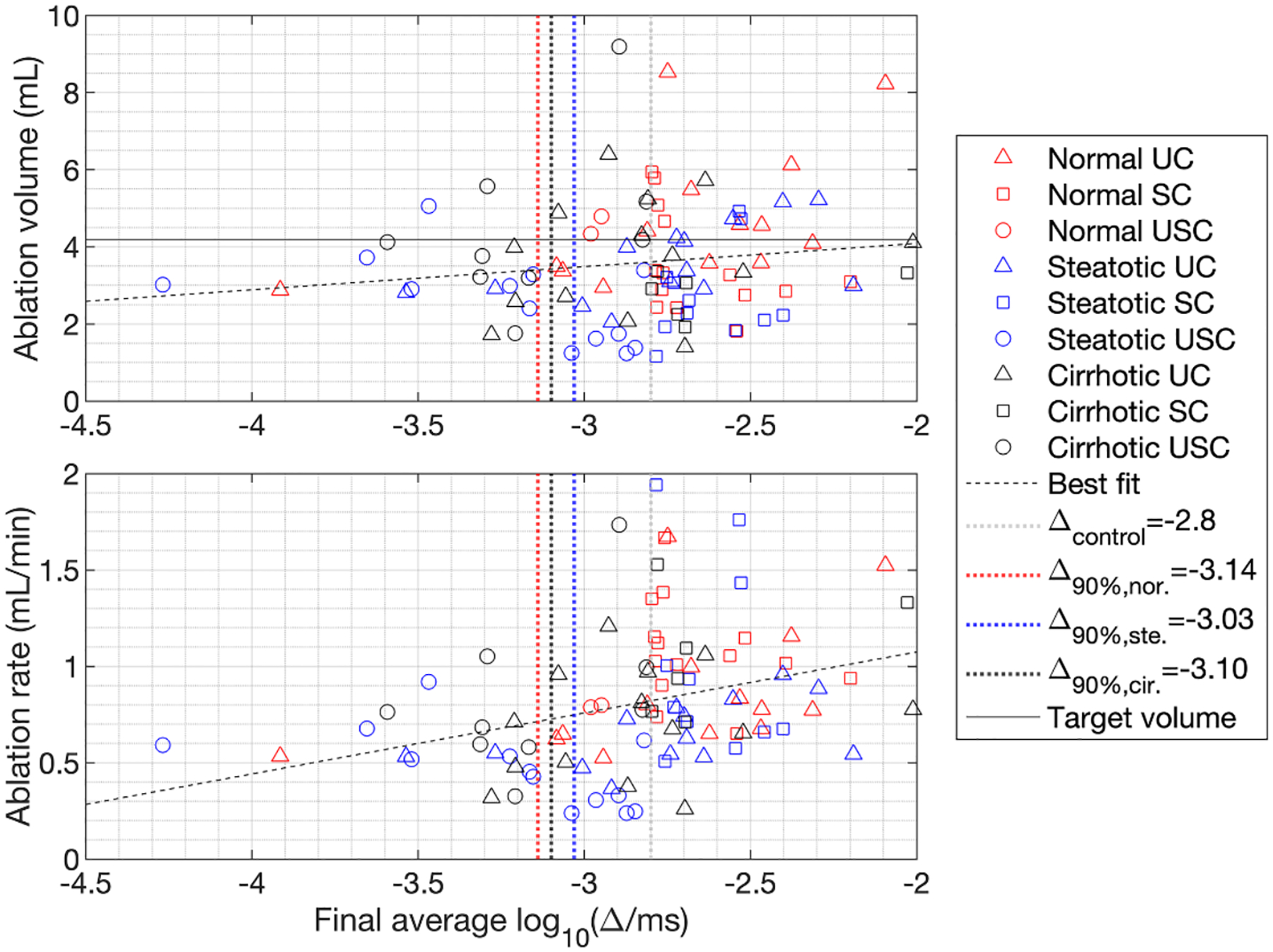
Scatter plots of measured ablation volume (top) and ablation rate (bottom) vs. log_10_-scaled average cumulative decorrelation in the spherical control ROI with 20 mm diameter. The control threshold is shown with a gray vertical line, cutoff thresholds corresponding to 90% specificity of local ablation prediction for each liver tissue condition are marked using red, blue, and black vertical dotted lines for normal, steatotic, and cirrhotic livers, respectively, and the targeted ablation volume corresponding to a 20 mm-diameter sphere is plotted as a black horizontal line in the top panel. Lines of best fit, obtained from linear regression all plotted data points, are shown with black dashes in both panels.

Figure 5 shows scatter plots of measured ablated volume and ablation rate vs. final average log_10_-scaled decorrelation inside the control ROI for all primary trials (N=97). Normal, steatotic, and cirrhotic liver conditions are denoted by red, blue, and black markers, respectively. Control conditions are indicated by triangles for UC, squares for SC, and circles for USC trials. By definition, all SC trials fall to the right of the gray vertical dotted line that at the control threshold $\Delta_{\text{control}}=-2.8$ (log_10_-scaled $\Delta \;{\text{ms}}^{-1}$). Volume of the spherical targeted ablation volume is 4.19 ml for diameter 20 mm, marked in the ablation-volume plot by a gray horizontal dotted line. Lines of best fit from linear regression analysis are shown with black dashes in both plots. Pearson correlation coefficients between ablated volumes and final average decorrelation were statistically insignificant for ablation volume vs. decorrelation (r=0.149, RMS error 1.50 ml, p=0.145) and for Dice coefficient (comparing measured vs. targeted ablation regions) vs. final echo decorrelation (r=0.102, RMS error 0.122, p=0.520), but were highly significant for ablation rate vs. decorrelation (r=0.331, RMS error 0.352 ml min^−1^, $p=9.3\times 10^{-4}$).

Figure 5 also shows thresholds corresponding to 90% specificity for prediction of local ablation by echo decorrelation, computed by ROC analysis across all control conditions in figure 3(d), are shown as vertical dotted lines with $\Delta_{90\%,\text{ N}}=-3.21$ for normal, $\Delta_{90\%,\text{ S}}=-3.10$ for steatotic, and $\Delta_{90\%,\text{C}}=-3.08$ for cirrhotic liver conditions. These thresholds correspond to sensitivity of 0.63 for normal, 0.52 for steatotic, and 0.48 for cirrhotic liver. If these thresholds had been used as stopping criteria for RFA control, the final average decorrelation for some of the USC trials would have surpassed the lower control threshold, so that control success rates would have risen from 87.5% to 100% for normal liver, from 45.8% to 70.8% for steatotic liver, and from 40.0% to 60.0% for cirrhotic liver tissue.

**Figure 6.**
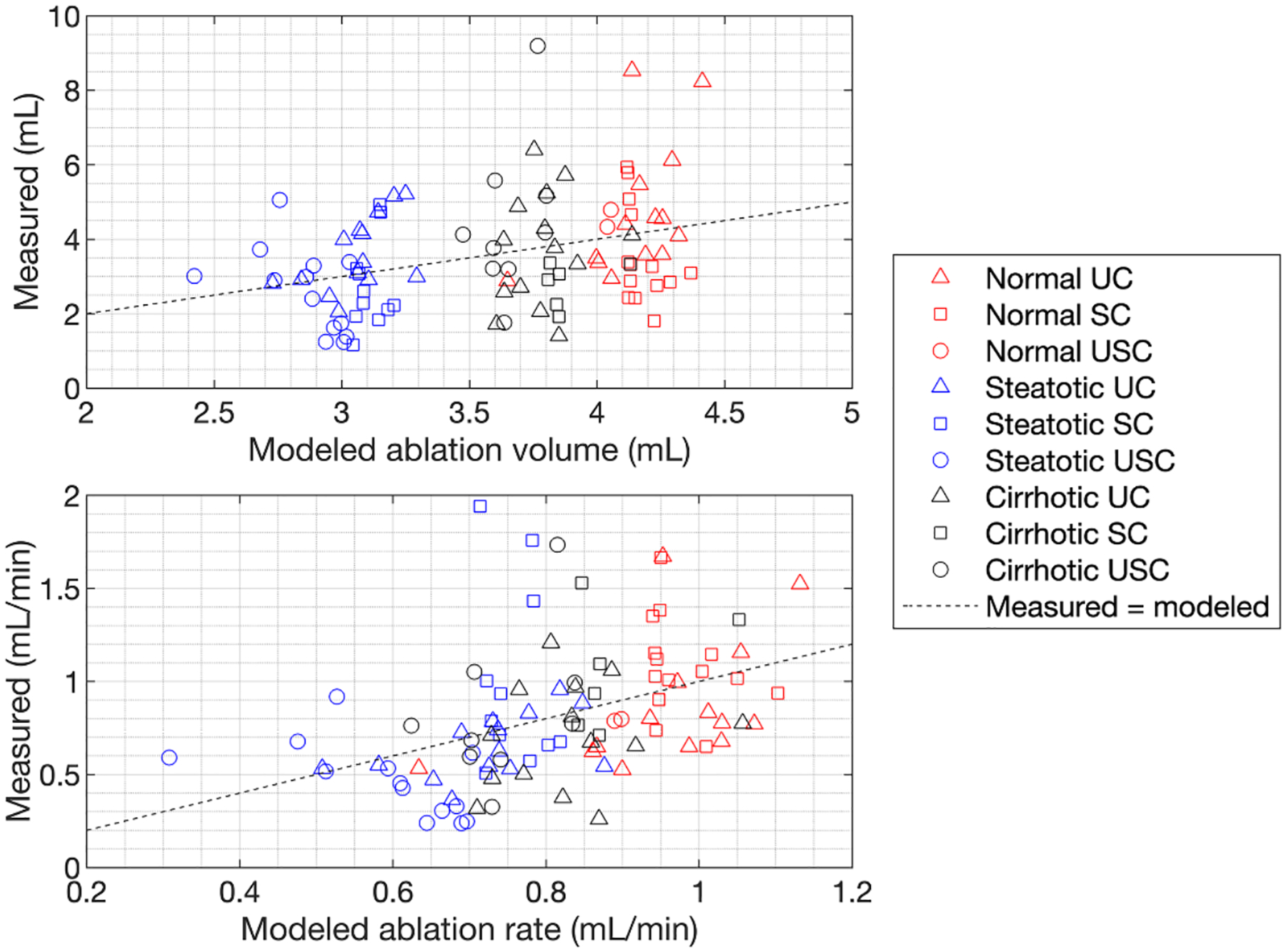
Scatter plots of measured ablation volume (top) and ablation rate (bottom) vs. output of a multiple linear regression model incorporating liver tissue condition as well as log10-scaled average cumulative decorrelation in the spherical control ROI. Lines corresponding to equality between predicted and measured outcomes are shown with black dashes in both panels.

Figure 6 shows results of multiple linear regression models for ablation volume and ablation rate based on final ROI-average cumulative echo decorrelation as well as tissue type. Models predicted ablation outcomes with high statistical significance for both ablation volume (r=0.339, RMS error 0.266 ml, $p=9.7\times 10^{-3}$) and ablation rate (r=0.423, RMS error 0.262 ml min^−1^, $p=3.6\times 10^{-4}$), but were not significant for prediction of the Dice coefficient between measured and targeted ablation regions (r=0.137, RMS error 0.102, p=0.624). In the multiple linear regression model predicting ablation volume, the only statistical significant individual contributor was the categorical variable for steatotis $\left(p=3.8\times 10^{-3}\right)$, while for the model predicting ablation rate, significant individual contributors included both steatosis $\left(p=6.9\times 10^{-3}\right)$ and decorrelation $\left(p=3.9\times 10^{-3}\right)$.

## 4. Discussion

This study has assessed feedback control using 3D echo decorrelation imaging to control RFA in healthy and diseased *ex vivo* human liver. The success rate of control (i.e. the fraction of controlled trials with final average decorrelation surpassing the prespecified threshold) was highest for normal liver tissue (87.5%), comparable to success rates previously seen for control by echo decorrelation in bulk ultrasound ablation of *ex vivo* bovine liver (77%) (Abbass *et al* 2018a), in HIFU ablation of *ex vivo* bovine liver (100%) (Abbass *et al* 2018b), in bulk ultrasound and HIFU ablation of *in vivo* rabbit liver and VX2 tumor (100%) (Abbass *et al* 2019), and for control of RFA in *ex vivo* bovine liver RFA using 3D echo decorrelation imaging (100%) (Grimm 2022). In contrast, control success rates were significantly smaller for steatotic (45.8%) and cirrhotic (40.0%) liver tissue.

In the present study, 3D echo decorrelation imaging was generally successful in predicting local ablation for RFA of human liver tissue. AUROC values observed were higher for all three control conditions, UC (AUROC ⩾ 0.916), SC (AUROC ⩾ 0.895), and USC (AUROC ⩾ 0.876) trials, compared to previous studies where 3D echo decorrelation imaging was used to monitor RFA (AUROC = 0.801) (Ghahramani *et al* 2022) or to control RFA (AUROC = 0.836 for SC and AUROC = 0.807 for UC trials) (Grimm 2022) of *ex vivo* bovine liver. Echo decorrelation was also found to correlate with ablation rate across all tissue types and control conditions, with high statistical significance. These results indicate that echo decorrelation imaging, previously validated for monitoring and control of thermal ablation in animal liver and tumor tissue both *ex vivo* and *in vivo* and for monitoring of clinical liver tumor ablation (Ghahramani 2024c), is also appropriate for monitoring and control of thermal ablation in normal and diseased human liver tissue.

This study provides new insight into differences in outcomes of both RFA and echo decorrelation imaging dependent on liver tissue condition. Comparing USC trials between the three tissue conditions, trials with steatotic liver resulted in smaller mean measured ablation volume, larger standard deviation for the Dice coefficient comparing measured versus targeted ablation zones, and smaller AUROC compared to normal or cirrhotic liver. These observations suggest that outcomes of RFA and echo decorrelation imaging in steatotic liver tissue are more variable than in cirrhotic or normal liver. In particular, the observed correspondence between echo decorrelation and local thermal ablation may be less robust for steatotic liver tissue than for normal or cirrhotic liver tissue. Consistent with these observations, the categorical variable representing steatosis was a highly significant contributor to multiple regression models for both ablation volume and ablation rate, providing further evidence that relationships between echo decorrelation and ablation outcomes depend strongly on physical properties of the tissue undergoing ablation.

Across all control conditions, the average ablated volume was largest for normal liver (4.16 ml, close to the targeted volume of a 20 mm diameter sphere), smaller for cirrhotic liver (3.77 ml), and smallest for steatotic liver tissue (3.00 ml). Similarly, in the multiple regression model for ablation volume, steatosis was the only statistically significant individual contributor. These results suggests that standard RFA exposure conditions designed for ablation of normal liver tissue may fall short for diseased liver tissue, particularly for steatotic liver. Increased fat deposits in steatotic liver may decrease tissue conductivity, resulting in smaller ablation volumes (Luo *et al* 2018). The smaller average ablation volume in cirrhotic liver tissue vs. the target volume could be partially due to vaporization of water content that is higher in cirrhotic liver, potentially resulting in greater heat-induced shrinkage compared to normal liver (Sy *et al* 2010).

The control threshold of −2.8 (log_10_-scaled Δ/ms) used here was chosen to achieve 90% specificity for prediction of local ablation by echo decorrelation in preliminary trials (N=12). For all trials considered here and separated by liver tissue condition (N⩾29 trials for each tissue condition), corresponding cutoff points for 90% specificity were smallest for normal liver tissue (−3.21, log_10_-scaled decorrelation per ms), larger for cirrhotic liver (−3.10), and largest for steatotic liver (−3.08). These cutoff points, which may be considered candidate thresholds for future implementations of thermal ablation control by echo decorrelation imaging, were generally lower compared to previous studies, such as thresholds similarly chosen or proposed for control of ultrasound ablation by 2D echo decorrelation imaging (−2.9 to −1.6, log_10_-scaled decorrelation per ms) (Fosnight *et al* 2017, Abbass *et al* 2018a, 2018b) as well as control of RFA by 3D echo decorrelation imaging in bovine liver tissue (−3.0 to −2.2) (Grimm 2022). Ordering of these cutoff points mirrored the ordering of average ablation volume for all control conditions (largest for normal and smallest for steatotic liver tissue), as shown in table 3. Because the log_10_-scaled average cumulative decorrelation within the control ROI, averaged across all control conditions, was not significantly different between liver tissue types, these trends suggest that higher control thresholds may be needed for control of RFA in cirrhotic and particularly in steatotic liver tissue, compared to normal liver. Use of tissue-specific control thresholds would be expected to increase rates of successful control, thus improving ablation outcomes for each liver tissue condition.

Modest differences in prediction performance of echo decorrelation imaging were seen when comparing between control conditions. For normal liver tissue, AUROC values were not significantly different between control conditions; for comparison, in previous studies of thermal ablation control in *ex vivo* bovine liver, AUROC for SC trials was found to be significantly better (Abbass *et al* 2018a, 2018b, Grimm 2022) or statistically equivalent (Fosnight *et al* 2017, Abbass *et al* 2018a, 2019), vs. UC trials. However, for diseased liver tissue, local ablation prediction performance showed different dependence on control conditions (in steatotic liver, significantly higher AUROC and AUPR for UC and SC trials compared to USC trials; in cirrhotic liver, significantly higher AUPR for UC and USC trials compared to SC trials). These results suggest that relationships between echo decorrelation and tissue ablation may differ between normal and diseased liver tissue.

In comparisons of ablation outcomes between control conditions, SC trials resulted in higher ablation rates for all three liver tissue conditions, but this difference was statistically significant only for SC vs. USC trials in steatotic liver. Ablation volumes showed no significant differences between control conditions for any tissue type. Consistent with previous studies of ablation control in *ex vivo* bovine liver, ablation rates were either significantly higher (Abbass *et al* 2019) or statistically equivalent (Abbass *et al* 2018b, 2019, Grimm 2022) for SC trials compared to UC trials, while ablation zone sizes were either significantly smaller (Abbass *et al* 2018a) or statistically equivalent (Abbass *et al* 2018b) for various ablation conditions. The generally higher ablation rate and smaller ablation size for SC trials, consistent with their intrinsically shorter ablation duration, suggest that control of clinical RFA by echo decorrelation imaging may help to minimize overtreatment of untargeted tissue, potentially reducing morbidity of such procedures.

The goal of more reproducible thermal ablation using control by echo decorrelation imaging was not fulfilled in this study. Average Dice coefficients comparing targeted versus measured ablation zones were generally lower for SC than for UC trials, though these differences were not statistically significant. In contrast, in a previous study of *ex vivo* bovine liver RFA control using 3D echo decorrelation imaging (Grimm 2022), Dice coefficients were significantly higher for SC (0.752) vs. UC (0.674) trials. Linear regression models relating these Dice coefficients to final ROI-average echo decorrelation and tissue condition were not statistically significant, indicating that spatially averaged echo decorrelation is not a strong predictor of conformity between targeted and measured ablation zones.

In a separate study (Ghahramani *et al* 2024b), we evaluated a deep learning-based segmentation method, 3D U-Net (Çiçek *et al* 2016), for the prediction of ablation zones using 3D echo decorrelation and B-mode image volumes using data from the first N=71 RFA trials reported here. Segmented 3D ablation zones were used as ground truth for training the convolutional neural networks. Results indicated that the 3D U-Net model, trained on the augmented 3D echo decorrelation image data, predicted the ablation volume significantly better (AUROC = 0.988, AUPR = 0.616) than simple thresholding of echo decorrelation maps using the cutoff corresponding to equal precision and recall (AUROC = 0.946, AUPR = 0.430; p<0.005). The resulting Dice coefficients comparing predicted versus measured ablation volumes were significantly higher for the 3D U-Net model (0.622 ± 0.121, mean ± standard deviation) compared to simple thresholding (0.396 ± 0.121, p<0.05).

Limitations of this study included the slice thickness for tissue sectioning (3–4 mm), which caused error in volumetric reconstruction of the ablation zone, as well as in registration error between B-mode image volumes and tissue sections. Using a smaller, more precisely controlled sectioning thickness for ablated liver specimens would decrease these errors. Moreover, expansion of liver tissue around the ablation zone, due to freezing the samples between ablation and sectioning, could affect the lesion shape and result in errors when comparing tissue sections with echo decorrelation maps. Another limitation of this study is the investigation of only normal, steatotic, and cirrhotic *ex vivo* liver tissue, without the presence of liver tumors. In future work, liver tissue samples including various types of hepatic tumor could be studied under similar protocols to verify the potential of 3D echo decorrelation imaging for control of liver tumor ablation. Use of a larger number of tissue samples than those employed here (e.g. N=97 specimens from 46 distinct donors in our primary trials) would enable more detailed investigation on the effects of lesion type and liver tissue conditions.

Further, this control approach should be tested and verified for thermal ablation of *in vivo* human liver tumors. Recent studies have shown feasibility for monitoring percutaneous thermal ablation of human liver tumor using 3D echo decorrelation imaging (Ghahramani 2024c, Abbass *et al* 2023) in clinical settings, though without closed-loop control. Because control of thermal ablation has been shown feasible using 2D echo decorrelation for an *in vivo* rabbit liver tumor model (Abbass *et al* 2019) and using 3D echo decorrelation for an *in vivo* swine liver model (Ghahramani *et al* 2024a), results of this study and these cited *in vivo* studies indicate control of clinical thermal ablation in human liver tumors using echo decorrelation imaging is likely also feasible.

To clinically translate 3D echo decorrelation imaging for control of thermal ablation, real-time computation of echo decorrelation imaging and a control GUI could be implemented on commercial ultrasound scanners, enabling real-time monitoring thermal ablation by displaying overlaid echo decorrelation on grayscale B-mode images, similar to figure 2. To mitigate artifacts from echo decorrelation induced by respiratory motion inherent to *in vivo* ablation, as well as by electronic noise, previously established methods can be used to remove estimated artifactual decorrelation from each frame (Hooi *et al* 2015, Fosnight *et al* 2017, Ghahramani 2024c). In these methods, motion-induced decorrelation is estimated from pre-ablation image data, then effectively subtracted from echo decorrelation maps throughout ablation. Effects of respiratory tissue motion on both echo decorrelation and image registration can be further mitigated *in vivo* by gating acquisition of echo decorrelation images to a specific point in each respiratory cycle (Ghahramani 2024c). These correction methods do not remove decorrelation associated with transient, heat-induced tissue motion such as that caused by tissue coagulation, contraction, or cracking; such heat-induced motion is instead a key contributor to the measured heat-induced echo decorrelation.

Predicted ablation zone boundaries could be delineated from echo decorrelation maps in real time using either thresholding or an appropriate machine-learning model, displayed on a clinical ultrasound scanner, and given integration with a thermal ablation system, employed for 3D control of the entire ablation zone. Because the present study has shown differences in thermal ablation and echo decorrelation outcomes dependent on tissue conditions, control thresholds for clinical ablation should be defined dependent on tissue type, including the type and location of tumor being ablated, as well as disease conditions of the surrounding parenchyma. For RFA, to achieve optimal outcomes, exposure settings should also be adjusted depending on tissue type, e.g. with progressively higher power in normal, cirrhotic, and steatotic liver tissue. Real-time monitoring and control by echo decorrelation imaging could be augmented by post-ablation contrast ultrasound, CT, or MR imaging to confirm completeness of ablation and assess the need for repeated treatment.

## 5. Conclusion

In this study, 3D echo decorrelation imaging feedback was employed to control RFA in normal and diseased *ex vivo* human liver parenchyma. Although local ablation was successfully predicted using 3D echo decorrelation imaging (AUROC ⩾ 0.876) for all liver types and control conditions, other results show substantial differences in outcomes of controlled and uncontrolled RFA and corresponding echo decorrelation. Ablations were successfully controlled in 87.5%, 45.8%, and 40.0% of controlled trials in normal, steatotic, and cirrhotic liver parenchyma, respectively, indicating a need for tissue-dependent control thresholds, such as those measured to correspond with 90% specificity for prediction of local ablation. Average measured ablated volumes for steatotic and cirrhotic liver samples were generally smaller than the targeted volume, while those for normal liver samples were near the targeted volume. Successfully controlled trials showed generally higher ablation rates and smaller ablation volumes compared to uncontrolled trials. Echo decorrelation was highly correlated with ablation rate across all liver types and control conditions. Multiple regression models incorporating echo decorrelation and tissue condition yielded highly significant predictions of ablation volume and rate, with steatotic condition contributing significantly to both models. These findings support the potential use of 3D echo decorrelation imaging feedback for monitoring and control of clinical thermal ablation of tumors within normal and diseased liver, while also indicating the value of accounting for liver tissue condition in such applications.

## Figures and Tables

**Table 1.** Demographic data for the liver samples used in preliminary and primary trials.

	Normal	Steatotic	Cirrhotic
	Preliminary trials		
Male/female	2/2	0/2	0/6
Age	64.5 ± 4.20	53 ± 0	53.33 ± 15.0
Race W/AA/H	2/2/0	2/0/0	2/4/0
	Primary trials		
Male/female	19/11	31/7	14/15
Age	53.5 ± 17.6	56.1 ± 7.61	58.4 ± 9.36
Race W/AA/H	21/6/3	31/7/0	27/2/0

W: White, AA: African American, H: Hispanic.

**Table 2.** Statistical analysis of ROC and PR curves for each group.

		ROC curves				PR curves		
		AUROC		$J_{\text{max}}$	Optimal threshold from $J_{\text{max}}\left[{\text{log}}_{10}\Delta \;{\text{ms}}^{-1}\right]$	AUPR	$F_{1\text{max}}$	Optimal threshold from $F_{1\text{max}},{\text{log}}_{10}\left(\Delta \right)\;{\text{ms}}^{-1}$
Groups	N	Across control conditions	Each group					
Normal UC	14	$\begin{array}{c} \;\\ \;\\ \; \end{array}\}0.922$	0.916	0.695	−4.083	0.423	0.494	−3.09
Normal SC	14		0.936	0.755	−4.30	0.387	0.460	−3.06
Normal USC	2		0.953	0.783	−5.43	0.634	0.613	−4.16
Steatotic UC	14	$\begin{array}{c} \;\\ \;\\ \; \end{array}\}0.921$	0.936	0.769	−3.93	0.350	0.456	−3.15
Steatotic SC	11		0.934	0.756	−3.87	0.399	0.466	−3.10
Steatotic USC	13		0.876	0.618	−4.20	0.164	0.259	−3.52
Cirrhotic UC	14	$\begin{array}{c} \;\\ \;\\ \; \end{array}\}0.907$	0.923	0.727	−3.79	0.328	0.407	−3.10
Cirrhotic SC	6		0.895	0.647	−3.47	0.214	0.361	−2.95
Cirrhotic USC	9		0.902	0.671	−3.92	0.335	0.441	−3.41

UC, SC, USC, N: number of cases, AUROC, AUPR, Jmax: maximum Youden index, F1max: maximum F1-score, SD: standard deviation.

**Table 3.** Mean ± standard deviation for measured ablation outcomes.

Groups	Ablation volume (ml)	Ablation rate (ml min^−1^)	Dice (ablation zone versus control ROI)	Final average ${\text{log}}_{10}\left(\Delta \right)$ in control ROI
Normal UC	4.70 ± 1.80	0.871 ± 0.353	0.602 ± 0.0973	−2.72 ± 0.446
Normal SC	4.55 ± 1.29	1.08 ± 0.261	0.550 ± 0.0802	−2.65 ± 0.184
Normal USC	4.56 ± 0.320	0.793 ± 0.00690	0.640 ± 0.0460	−2.96 ± 0.0226
Steatotic UC	3.58 ± 1.00	0.649 ± 0.172	0.604 ± 0.0970	−2.75± 0.361
Steatotic SC	2.74 ± 1.18	0.999 ± 0.493	0.528 ± 0.125	−2.62 ± 0.134
Steatotic USC	2.62 ± 1.14	0.469 ± 0.202	0.494 ± 0.180	−3.22 ± 0.415
Cirrhotic UC	3.73 ± 1.52	0.697 ± 0.288	0.583 ± 0.114	−2.85 ± 0.333
Cirrhotic SC	2.81 ± 0.593	1.06 ± 0.322	0.487 ± 0.0897	−2.62 ± 0.292
Cirrhotic USC	4.46 ± 2.10	0.834 ± 0.402	0.599 ± 0.0887	−3.16 ± 0.263

UC, SC, USC, ROI: region of interest, Δ: echo decorrelation.

## Data Availability

The data that support the findings of this study are openly available from the site scholar.uc.edu at the address https://doi.org/10.7945/2g9q-t294
